# Analysis of Therapeutic Targets of A Novel Peptide Athycaltide-1 in the Treatment of Isoproterenol-Induced Pathological Myocardial Hypertrophy

**DOI:** 10.1155/2022/2715084

**Published:** 2022-05-02

**Authors:** Xi Zheng, Fuxiang Su, Ze Kang, Jingyuan Li, Chenyang Zhang, Yujia Zhang, Liying Hao

**Affiliations:** ^1^Department of Pharmaceutical Toxicology, School of Pharmacy, China Medical University, Shenyang 110122, China; ^2^Department of Cardiology, Shengjing Hospital, China Medical University, Shenyang 110020, China; ^3^Department of Clinical Pharmacy, The First Affiliated Hospital of Jinzhou Medical University, Jinzhou 121001, China

## Abstract

Myocardial hypertrophy is a pathological feature of many heart diseases. This is a complex process involving all types of cells in the heart and interactions with circulating cells. This study is aimed at identifying the differentially expressed proteins (DEPs) in myocardial hypertrophy rats induced by isoprenaline (ISO) and treated with novel peptide Athycaltide-1 (ATH-1) and exploring the mechanism of its improvement. ITRAQ was performed to compare the three different heart states in control group, ISO group, and ATH-1 group. Pairwise comparison showed that there were 121 DEPs in ISO/control (96 upregulated and 25 downregulated), 47 DEPs in ATH-1/ISO (27 upregulated and 20 downregulated), and 116 DEPs in ATH-1/control (77 upregulated and 39 downregulated). Protein network analysis was then performed using the STRING software. Functional analysis revealed that Hspa1 protein, oxidative stress, and MAPK signaling pathway were significantly involved in the occurrence and development of myocardial hypertrophy, which was further validated by vivo model. It is proved that ATH-1 can reduce the expression of Hspa1 protein and the level of oxidative stress in hypertrophic myocardium and further inhibit the phosphorylation of p38 MAPK, JNK, and ERK1/2.

## 1. Introduction

Myocardial hypertrophy is one of the main risks of heart failure, which is characterized by abnormal enlargement of myocardium [1]. It is due to the increase of myocardial cell size and nonmuscle cell proliferation [2]. Potential causes of myocardial hypertrophy include hypertension, cardiomyopathy, valvular insufficiency, and myocardial infarction [3]. Sympathetic activation is closely related to cardiac function, and *β* adrenergic receptors are considered to be important parts of sympathetic activation [4]. Isoprenaline (ISO), as an agonist of *β* adrenergic receptor, can simulate sustained adrenergic stimulation and develop into maladaptive myocardial hypertrophy [5]. Calmodulin (CaM), Ca^2+^/calmodulin-dependent protein kinase II (CaMKII), and L-type calcium channel (LTCC) play an important role in the occurrence and development of myocardial hypertrophy [6, 7]. LTCC contains four subtypes from Ca_V_1.1 to Ca_V_1.4, and Ca_V_1.2 is highly expressed in myocardial tissue [8]. Our previous study showed that CaMKII-induced phosphorylation of Thr1604 residue in Ca_V_1.2 channel may be one of the significant characteristics of myocardial hypertrophy and heart disease [9]. Based on these results, we designed a novel peptide Athycaltide-1 (ATH-1), which can restore the normal level of hypertrophic myocardium. The amino acid sequence of ATH-1 is GRKKRRQRRRGGDDEVTVGKFYATFLIQEYFRKFKKRKEQGL. Its structure is similar to Ca_V_1.2 channel protein, which can affect the binding of Ca_V_1.2 channel protein to CaMKII and CaM. However, the specific mechanism of how ATH-1 works in the process and development of improving myocardial hypertrophy induced by ISO is not clear. Due to the incompleteness of the aforementioned studies, we need to identify the differentially expressed proteins (DEPs) and find targets to explore the mechanism.

The combination of absolute quantification (iTRAQ) and liquid chromatography tandem triple quadrupole time-of-flight high resolution mass spectrometry (LC-TripleTOFMS/MS) is a highly sensitive and practical method for identifying various disease targets and has been widely used [10]. We used normal rat hearts, hypertrophic rat hearts induced by ISO, and the rat hearts recovered from hypertrophy after treatment with ATH-1 to compare and quantitatively analyze the cardiac proteome. iTRAQ technology combined with LC-TripleTOFMS/MS was applied to identify the DEPs in the three groups, and the target proteins and key pathways were screened for verification, so as to preliminarily explore the mechanism of recuperating from myocardial hypertrophy by treating with ATH-1.

## 2. Materials and Methods

### 2.1. Animals

Healthy male SD rats weighing 180-220 g (Liaoning Changsheng Biotechnology Co., Ltd., Liaoning, China, SCXK (Liao) 2015-0001) were reared under standard conditions (temperature 21 ± 1°C and humidity 55-60%). The rats were acclimated for one week before the experiment. Food and water were provided to rats freely. The procedure was in accordance with the provisions of the Animal Protection and Use Committee of China Medical University. The rats used for proteomic detection were divided into three groups. The control group rats were kept in the same environment as other groups for 3 weeks. In the ISO group, after one week of normal feeding, ISO (Sigma, Switzerland) was injected subcutaneously at the rate of 5 mg/kg body weight every day for 2 weeks. In the ATH-1 group, 5 *μ*g/kg/day ATH-1 was injected intraperitoneally for the first week. Then, 5 mg/kg/day ISO and 5 *μ*g/kg/day ATH-1 were injected together for the last 2 weeks.

In the validation model of myocardial hypertrophy treated with ATH-1, the rats were randomly divided into three groups: control group, ISO group, and ATH-1 group. The control group rats were kept in the same environment as other groups for 5 weeks. Rats in the ISO group were injected subcutaneously with 5 mg/kg/day ISO (Sigma, Switzerland) for 2 weeks, and normal saline was injected for the remaining 3 weeks. Rats in the ATH-1 group were injected subcutaneously with 5 mg/kg/day ISO for 2 weeks and then injected intraperitoneally with 5 *μ*g/kg/day ATH-1 for 3 weeks. Each group of rats was euthanized at the end of the fifth week, and then, the hearts were collected.

### 2.2. Histopathological Analysis

The rat myocardial tissues underwent fixation with 4% formalin (4 h), paraffin embedding, and sectioning at a thickness of 5 *μ*m. After dewaxing with xylene and rehydration through graded ethanol, the specimens underwent staining with hematoxylin and eosin (HE) and Masson's Trichrome Stain Kit.

### 2.3. iTRAQ Sample Labeling

The sample was mixed with cold acetone (99.5%) at -20°C for 2 h and centrifuged at 4°C and 3000 × g for 5 min to precipitate protein. After air drying, the precipitates were dissolved in UA (8 M urea and 0.1 M Tris HCl, pH 8.5). The protein concentration was determined by Bradford method; then, the protein was alkylated and digested with trypsin at 37°C for 12 h (each sample 200 *μ*g). The samples were labeled with different isobaric labeling reagents (iTRAQ8plex reagent and AB-Sciex reagent) and collected.

### 2.4. Strong Cation Exchange Chromatography

The mixed sample was added to SCX buffer A (20 mM ammonium formate, pH 10.0) and mixed well. After centrifugation at 11000 × g and 4°C for 5 minutes, iTRAQ-labeled peptides were separated by HPLC 2010A system (Shimadzu Corporation) and Gemini NX column (4.6 × 250 mm, 5 *μ*m, 110 Å, Phenomenex, PN:00G-4454-E0). The linear gradient elution of 0-20% SCX buffer A containing 20 mM ammonium formate for 60 minutes was followed by gradient elution of 20-100% SCX buffer B containing 80% acetonitrile for 60 minutes (the flow rate was 0.8 ml/min at 25°C). The fractions were collected at 1 minute intervals and lyophilized in vacuum.

### 2.5. LC-TripleTOFMS/MS Analysis

The SCX component was resuspended in reversed-phase buffer A (98% H2O, 2% acetonitrile, and 0.1% formic acid). Then, the peptide was separated on a reversed-phase nano LC-Chrom XP-C18 column (chromxp-C18, 350 *μ*m × 0.5 mm, 3 *μ*m, 120 Å) with 60 minutes of linear gradient buffer A (0.1% formic acid in 2% acetonitrile) and buffer B (0.1% formic acid in 98% acetonitrile) at 300 m*μ*l/min. The mass spectrometry was carried out in the triple 5600 instrument (AB Sciex, LLC). Electrospray ionization (2.3 kV ion spray voltage) was used to produce positive ion quadrupole time of flight mass spectrometer (Q-TOF-MS) in an information-dependent acquisition mode (scanning range 350-1500 m*/z*, cumulative time 0.5 s). Finally, peptides were selected for MS/MS analysis.

### 2.6. Bioinformatics Analysis

Proteins meeting the following criteria were selected for further analysis: the expression was significantly changed (compared with the control group, the fold change ≥ 1.2 or ≤0.83, *P* value < 0.05); at least three peptides had 95% confidence and 1% of total FDR. Bioinformatics analysis ensured that all identified protein materials were submitted to the database for annotation, visualization, and comprehensive discovery (DAVID, Version 6.8, http://david.abcc.ncifcrf.gov). Then, the default matched organism was selected as the “background to be done” analysis. Functional annotation and enrichment analysis were performed for Gene Ontology (GO) annotation, biological process (BP), cellular component (CC), and molecular function (MF). The KEGG database (http://www.genome.jp/kegg/) was used to classify the identified proteins. The addition of differentially expressed proteins (DEPs) was submitted to the search tool (STRING v11.0, http://www.string-db.org/) for protein-protein interaction (PPI) files.

### 2.7. Total Superoxide Dismutase (T-SOD) Activity

The kit was purchased from Nanjing Jiancheng Biology Co., Ltd. Add every reagent application solution into the determination tube and control tube, respectively. 0.05 ml tissue homogenate and 0.05 ml distilled water were added to the test tube and control tube, respectively. The samples were thoroughly mixed with vortex mixer and placed in 37°C constant temperature water bath for 40 min. Then, 2 ml of developer was added into the samples and mixed well. Finally, the measurement was carried out at 550 nm wavelength.

### 2.8. Western Blot Assay

Myocardial tissue was homogenized in lysis buffer containing PMSF and protein kinase inhibitor. After vortexing, the lysate was centrifuged at 13500 rpm/min for 15 min at 4°C and SDS-PAGE was performed. The protein samples separated by SDS-PAGE were then transferred to polyvinylidene fluoride (PVDF) membrane. The membrane was sealed with 5% BSA for 2 h at room temperature and incubated with anti-ERK1/2 (Cell Signaling Technology, #9926, 1 : 1000), anti-JNK (Cell Signaling Technology, #9926, 1 : 1000), anti-p38 MAPK (Cell Signaling Technology, #9926, 1 : 1000), anti-p-ERK1/2 (Cell Signaling Technology, #9910, 1 : 1000), anti-p-JNK (Cell Signaling Technology, #9910, 1 : 1000), anti-p-p38 MAPK (Cell Signaling Technology, #9910, 1 : 1000), anti-ANP (Abcam, ab181242, 1 : 10000), anti-BNP (Abcam, ab236101, 1 : 1000), and anti-GAPDH (Proteintech, 10494-1-AP, 1 : 10000) antibody overnight at 4°C. Goat anti-rabbit IgG (Cell Signaling Technology, #9910, 1 : 1000) was used to block for 2 h, and the membrane was washed three times. ECL was used to visualize these bands. The ImageJ software (http://rsb.info.nih.gov/ij/) was used for densitometry analysis.

### 2.9. Statistical Analysis

We used one-way ANOVA to count the differences between the three groups and used Bonferroni to further perform multivariate analysis of differences. All dates were expressed as the mean ± SEM. *P* < 0.05 was designated to be of statistical significance. Statistical analysis was performed using GraphPad Prism 5.

## 3. Results

### 3.1. Ameliorative Effect of ATH-1 on Myocardial Hypertrophy Induced by ISO

**Figure 1:**
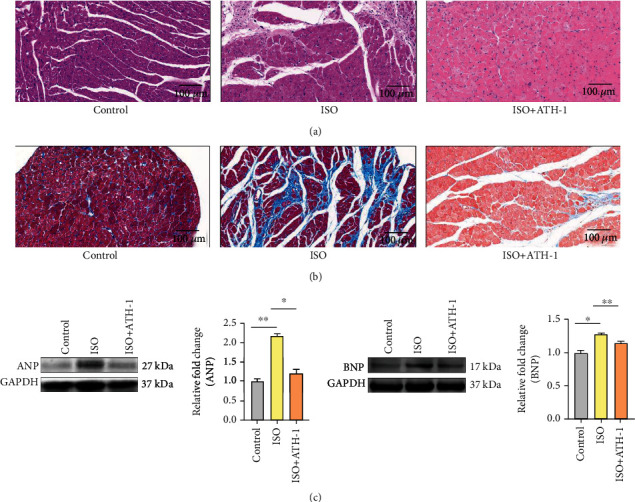
HE staining, Masson staining, and protein expression of ANP and BNP. (a) The cross-section of the cardiac tissue (the magnification is 20.0x) by HE staining in each group (*n* = 3). (b) The cross-section of the cardiac tissue (the magnification is 20.0x) by Masson staining in each group (*n* = 3). (c) The protein expression of ANP and BNP in each group (*n* = 3). ^∗^*P* < 0.05 and ^∗∗^*P* < 0.01.

We constructed the rat model of preventive administration of ATH-1. The HE staining results showed that the myocardial cross-sectional area upon ISO administration was significantly increased compared with those in rats treated with ATH-1 (Figure 1(a)). Consistent with these results, Masson staining in ISO-induced heart tissue showed more intense signals compared with the case in ATH-1-treated heart samples (Figure 1(b)). In addition, ANP and BNP were markedly increased in the ISO group compared with those in the ATH-1 group (Figure 1(c)). These results preliminarily confirmed the protective effect of ATH-1 on myocardium and could reverse myocardial hypertrophy.

### 3.2. Identification of DEPs

**Figure 2:**
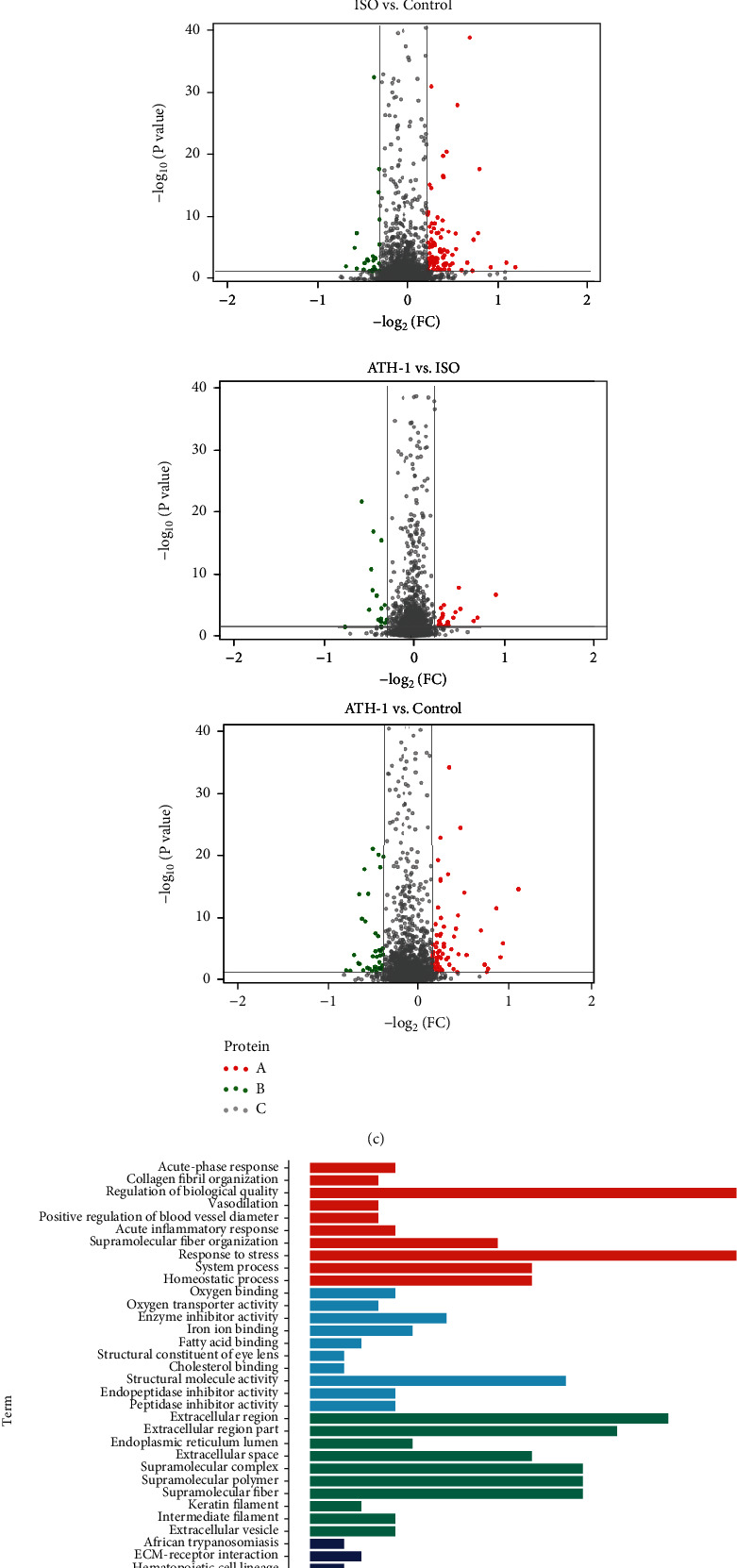
(a) Histogram of upregulated and downregulated DEPs. Upregulated DEPs were in grey, and downregulated DEPs were in yellow. (b) The heatmap and cluster analysis of DEPs. Upregulated DEPs were in red, and downregulated DEPs were in green. (c) Volcano plot representation of DEPs. A: upregulated proteins; B: downregulated proteins; and C: unchanged proteins. (d) GO enrichment and KEGG analysis of DEPs in the ISO/control and (e) ATH-1/ISO comparisons. The top 10 terms of BP, MF, CC, and KEGG analysis were displayed with the parameter protein count and *P* value. (f) Mapping of DEPs onto a composite network based on predicted protein-protein interaction. A predicted PPI network has been visualized for the identified DEPs.

We found that 121 protein expression levels were significantly changed in the ISO group comparing with the control group (*P* < 0.05), of which 96 protein expression levels were generally upregulated and 25 protein expression levels were generally downregulated by iTRAQ peptide labeling strategy (Figure 2(a)). There were 27 upregulated proteins and 20 downregulated proteins in ATH-1 vs. ISO comparison, and a total of 47 protein expression levels showed significant changes (*P* < 0.05) (Figure 2(a)). Compared with the control group, 77 proteins were upregulated and 39 proteins were downregulated in the ATH-1 group (*P* < 0.05) (Figure 2(a)). Heatmap and volcano plots showed the total number of upregulated and downregulated DEPs (Figures 2(b) and 2(c)).

### 3.3. Bioinformatics Analysis of DEPs

In order to further understand the function of DEPs, we performed GO and KEGG enrichment analysis. The top 10 biological processes, cellular components, molecular functions, and pathways were identified. In the ISO/control comparison, 4 biological processes were related to myocardial hypertrophy (“vasodilation,” “positive regulation of blood vessel diameter,” “acute inflammatory response,” and “response to stress”), and the other 6 were related to metabolic processes. In terms of molecular functions, the most important GO terms were “oxygen binding,” “oxygen transporter activity,” and “enzyme inhibitor activity.” In terms of cell components, it mainly involved extracellular components and cytoskeleton. KEGG pathway enrichment analysis showed that in myocardial hypertrophy induced by ISO, it mainly played a role in pathways about “ECM receptor interaction,” “complex and coagulation cascades,” “hypertrophic cardiomyopathy (HCM),” “phagosome,” “differentiated cardiomyopathy,” and “regulation of actin cytoskeleton” (Figure 2(d)).

In the ATH-1/ISO comparison, the biological processes corresponding to DEPs were mainly related to metabolic processes and regulation of immune response. In terms of molecular functions, the most important GO terms were “oxygen binding,” “oxygen transporter activity,” and “carbon dehydrogenase activity.” In terms of cell components, it mainly involves organelles and cytoskeleton. KEGG pathway enrichment analysis showed that 116 DEPs significantly enriched in 36 signaling pathways, which were closely related to “T cell receptor signaling pathway,” “nitrogen metabolism,” “primary immune deficiency,” “regulation of autophagy,” “MAPK signaling pathway,” and “cell adhesion molecules (CAMs)”(Figure 2(e)).

### 3.4. PPI Network Analysis

In order to visualize the interaction between DEPs, STRING database was used to build the interaction network for disordered DEPs (Figure 2(f)). We found that 123 DEPs formed a complex interaction network, including 123 nodes and 227 edges. The average node degree was 3.69, and the clustering coefficient was 0.494. The expected number of edges for this analysis was 74. Moreover, the PPI enrichment *P* value was less than 1.0 × 10^−16^, which indicated that the DEPs were at least partially biologically connected as a group.

### 3.5. Validation of DEPs and Signaling Pathways

**Figure 3:**
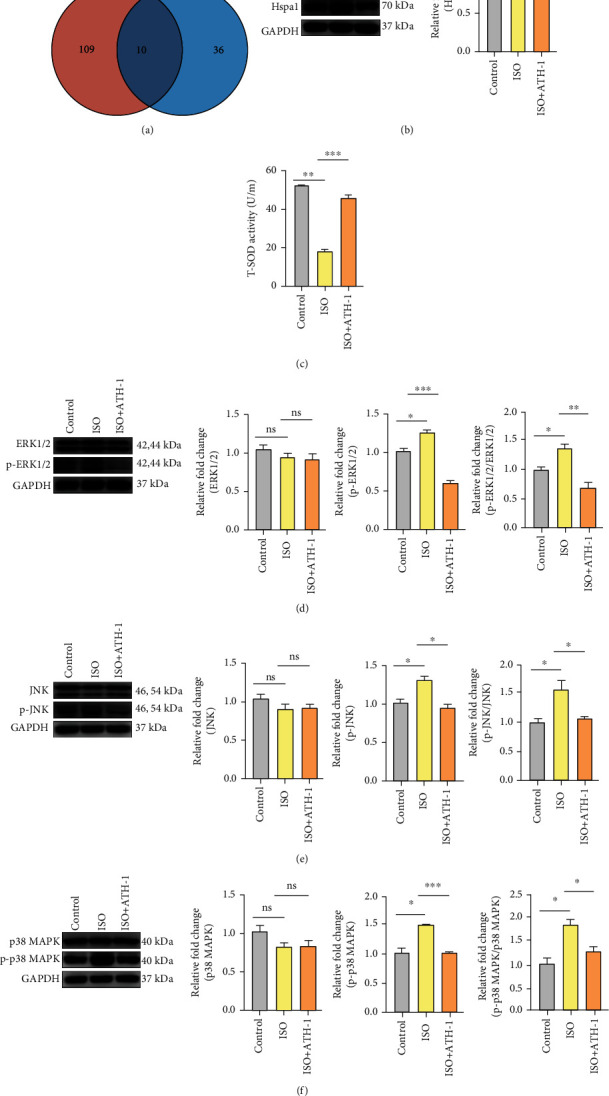
ATH-1 decreased the protein expression of Hspa1, p-ERK, p-JNK, and p-p38 in hypertrophic myocardium and enhanced the activity of T-SOD. (a) Differentially abundant proteins identified in the iTRAQ experiment. (b) The protein expression of Hspa1 (*n* = 3). ^∗^*P* < 0.05 and ^∗∗^*P* < 0.01. (c) The activity of T-SOD in each group (*n* = 3). ^∗∗^*P* < 0.01 and ^∗∗∗^*P* < 0.001. (d) The protein expression of ERK1/2 and p-ERK1/2 (*n* = 3). ns indicates none significance, ^∗^*P* < 0.05, ^∗∗^*P* < 0.01, and ^∗∗∗^*P* < 0.001. (e) The protein expression of JNK and p-JNK (*n* = 3). ns indicates none significance, ^∗^*P* < 0.05. (f) The protein expression of p38 MAPK and p-p38 MAPK (*n* = 3). ns indicates none significance, ^∗^*P* < 0.05 and ^∗∗∗^*P* < 0.001.

To verify the iTRAQ results, we constructed ATH-1 treatment models. We focused on the DEPs with significant changed in both ISO vs. control and ATH-1 vs. ISO, and a total of 10 DEPs were found (Figure 3(a)). The information of these 10 DEPs is shown in Table 1. We found that Hspa1 had the most unique peptides among the 10 DEPs, which was quantified by western blot. The results showed that the expression of Hspa1 in the ISO group was significantly higher than that in the control group and compared with the ISO group that was evidently lower in the ATH-1 group (Figure 3(b)). In addition, based on GO and KEGG analysis of ISO/control and ATH-1/ISO, we speculated that the improvement of myocardial hypertrophy by ATH-1 was closely related to the level of oxidative stress and MAPK signaling pathway. We detected the activity of T-SOD by the kit to assess the level of oxidative stress. The results showed that the activity of T-SOD in the ISO group was significantly lower than that in the control group, and the activity of T-SOD in the ATH-1 group was higher than that in the ISO group (Figure 3(c)). Western blot quantification of the MAPK signaling pathway showed that p-p38 MAPK, p-JNK, and p-ERK1/2 in the ISO group were phosphorylated and activated. But they were inhibited after ATH-1 administration (Figures 3(c)–3(e)).

## 4. Discussion

Myocardial hypertrophy is thought to be related to a variety of pathological conditions, including hypertension, vascular disease, and chronic heart failure [11]. It is considered to be a maladaptive response to stress or volume overload, leading to the activation of hypertrophic genes and signaling pathways [12, 13]. The development targets for detection and treatment of myocardial hypertrophy mainly focus on micro-RNAs, vascular endothelial growth factor, NAD-dependent deacetylase sirtuin-3 (SIRT3), growth/differentiation factor 15 (GDF15), transmembrane protein glycoprotein 130 (Gp130), CaMKII, Chloride channel-3 (ClC-3), and adhesion molecules [14]. CaMKII is considered to be a node influencing factor of excitation-contraction and excitation-transcription coupling and has long been regarded as a promising target for pharmacological inhibition. However, it is difficult to target the site of action because of the universality of CaMKII [15]. The novel peptide ATH-1 we designed can affect the binding of Ca_V_1.2 channel protein with CaMKII and CaM. Considering the ubiquity of CaMKII, it is unknown whether ATH-1 will act on other targets and signaling pathways in the diseased heart. Quantitative analysis through iTRAQ has high reproducibility and low interexperimental differences and has recently been proposed as a suitable method for detecting target proteins [16]. In this study, we used a combination of iTRAQ and LC-MS/MS to analyze the proteome of the normal myocardial tissue, ISO-induced cardiac hypertrophy tissue, and preventive administration of ATH-1 myocardial tissue. We identified a total of 168 DEPs, of which there were 121 significant DEPs in the control group/ISO group (96 upregulated and 25 downregulated) and 47 DEPs in the ATH-1 group/ISO group (27 upregulated and 20 downregulated). Functional analysis determined the main functions of proteins that change oxidative stress, immune response regulation, and cytoskeleton during ISO-induced myocardial hypertrophy and ATH-1 treatment. Pathway analysis showed that T cell receptor signaling pathway, regulation of autophagy, and MAPK signaling pathway may play an important role in the development of ATH-1 in the treatment of ISO-induced myocardial hypertrophy. We observed a total of 10 proteins with significant changed in both ISO vs. control and ATH-1 vs. ISO. Among these DEPs, Hspa1 has the most unique peptides in the ATH-1 group/ISO group. Therefore, we preliminarily studied whether the effect of ATH-1 in the treatment of myocardial hypertrophy is related to Hspa1, oxidative stress, and MAPK signaling pathway.

Heat shock protein (Hsp) is a large molecular chaperone protein that can regulate the balance of protein synthesis and degradation, assist in the refolding of misfolded proteins, and prevent cell death when induced under stress/pathological conditions [17, 18] and indirectly affects protein degradation [19] and DNA repair in the nucleus and nucleolus [20]. Hsp is divided into different families based on molecular size and amino acid sequence similarity, the largest of which is Hspa [21]. Hspa1 (also names Hsp70) and Hspa2 are the main members of the Hspa family. Hspa1 is expressed at low levels in normal cells with low stress and is highly activated under high stress stimulation and a wide range of physiological and pathological conditions [22]. Pathological myocardial stress (such as hypertrophy or ischemia) will increase the number of misfolded protein [23–25]. In order to counteract this stress and the accumulation of misfolded proteins during myocardial ischemia, the expression of Hsp70 can be rapidly induced [26]. Elevated levels of myocardial Hsp70 can reduce infarct size and improve recovery after ischemia [27–29]. The increase of Hsp70 level also helps to enhance cell protection and the ability to resist stress. In addition, Hsp70 is also involved in the regulation of immune and inflammatory responses in the process of tissue injury [30, 31] and also exhibits brain protection in focal cerebral ischemia [32]. Therefore, increasing Hsp therapy will benefit the heart's protective effect under stress and guide the development of drugs [33, 34]. Although Hspa1 has been proved to have an important protective effect on acute myocardial ischemic injury, more and more studies have shown that long-term overexpression of Hspa1 can aggravate myocardial remodeling and lead to heart failure. Extracellular Hsp70 has been identified as an independent prognostic marker of death in patients with heart failure [35] and has been found to be an independent prognostic marker of patients with cardiac arrest [36]. Yoon et al. [37] found that the phosphorylation of histone deacetylase 2 (HDAC2) induced by hypertrophic stress enhances the specific binding of HDAC2 to Hsp70 and maintains its phosphorylation state, leading to the activation of HDAC2 and the development of myocardial hypertrophy. Therefore, Hsp70 blockers are associated with disease modulators of target protein phosphorylation will be a new strategy for the treatment of heart diseases. Higher Hsp70 levels can also be observed in pulmonary artery smooth muscle cell (PASMC) with pulmonary hypertension (PAH). This upregulation can promote the expression of pho-I*κ*B*α*, which is a NF-*κ*B signaling pathway inhibitor. Upregulation of the NF-*κ*B signaling pathway can promote PASMC proliferation and pulmonary vascular remodeling in PAH, leading to increased pulmonary pressure and subsequent right heart failure caused by right ventricular hypertrophy [38]. Extracellular Hsp70 significantly contracted left ventricular dilation and dysfunction caused by doxorubicin and also significantly promoted cardiac fibrosis. The cardioprotective effect of anti-Hsp70 antibody is mainly attributed to its ability to promote the regression of myocardial inflammation, such as the subunit of nuclear factor-*κ*B as evidenced by its inhibition of toll-like receptor 2-related signal cascade and regulation of the intracellular distribution of p50 and p65 [39]. Zerikiotis et al. [40] found that the concentration of Hsp70 in the serum of patients with heart failure increased, and Hspa1 may be an important clinical biomarker for evaluating the severity of heart failure. It is worth noting that long-term overexpression of Hsp70 cannot prevent cardiac dysfunction and adverse remodeling after chronic heart failure and atrial fibrillation [41]. Therefore, inhibiting the long-term overexpression of Hspa1 may be a new treatment strategy for myocardial hypertrophy.

Oxidative stress refers to high levels of reactive oxygen species (ROS) in cells, which can cause damage to lipids, proteins, and DNA [42]. More and more evidences showed that oxidative stress is an important factor leading to myocardial hypertrophy [43]. However, under pathological conditions such as heart failure and ischemia/reperfusion, ROS levels may increase and make the heart prone to arrhythmia. At the same time, oxidative stress leads to the activation of CaMKII and the disturbance of intracellular ion homeostasis [44]. There are many cellular intermediate products affected by ROS changes but mainly include ion channels and their regulators [45]. The overproduction of ROS can change the activation of mitogen-activated protein kinases (MAPKs), protein kinase B (PKB), and calcineurin. These protein kinases can regulate the subsequent development of myocardial hypertrophy [46]. Therefore, inhibiting the level of oxidative stress may improve cardiac remodeling.

As a multifunctional regulator, MAPKs play an important role in regulating myocardial hypertrophy and myocardial remodeling to cope with increased load or pathological damage [47]. The activation of MAPKs is usually achieved through membrane receptors or other pressure-sensitive effectors and then activates a series of highly ordered protein kinase sequences that can amplify signals and mediate different cytoplasm and nuclei and regulate the phosphorylation of proteins [48]. The MAPK signaling cascade finally activates p38 MAPK, c-Jun N-terminal kinases (JNK), or extracellular signal-regulated kinases (ERK) to regulate cell proliferation, differentiation, apoptosis, growth, cell recombination, and metabolism [49]. In end-stage heart failure or pathological myocardial hypertrophy, almost all MAPK signal components are activated, and their phosphorylation drives various effectors [50, 51].

## 5. Conclusions

Our results show that ATH-1 can inhibit the expression of Hspa1 in the body of ISO-induced pathological myocardial hypertrophy. ATH-1 also improved myocardial oxidative stress to a certain extent and affected the activation of MAPK signaling pathway. In the state of ISO-induced cardiac hypertrophy, p38 MAPK, JNK, and ERK1/2 are all activated by phosphorylation, while ATH-1 significantly reduces the phosphorylation of MAPKs and inhibits their activation, thereby improving the state of myocardial hypertrophy. However our research is only based on the analysis of iTRAQ, and further verification is needed. In addition, the verification of clinical samples is needed to further test the effectiveness and safety of ATH-1.

## Figures and Tables

**Table 1:** DEPs with significant changed in both ISO vs. control and ATH-1 vs. ISO.

Accession	Gene name	Description	ISO vs. control *P* value	ATH-1 vs. ISO *P* value
P0DMW0	Hspa1	Heat shock 70 kDa protein 1A	3.26*E*-18	7.72 × 10^−5^
P11517	LOC689064	Hemoglobin subunit beta-2	8.65*E*-122	2.38*E*-17
A0A0G2JSV6	Hba2	Globin c2	0	7.57*E*-69
Q63011		Zero beta-globin (fragment)	6.73*E*-77	5.41*E*-08
A0A0G2JV08	Suz12	Suppressor of zeste 12 homolog (Drosophila)	3.59 × 10^−2^	4.14 × 10^−2^
F1LZH0	ENSRNOG00000049299	Ig kappa chain V19-17-like	5.08 × 10^−3^	6.65 × 10^−3^
P02770	Alb	Serum albumin	1.05*E*-09	3.61*E*-22
A0A0G2JSW3		Globin a4	1.23*E*-294	2.19*E*-54
F7FFV2	Krt5	Keratin, type II cytoskeletal 5 (fragment)	2.13 × 10^−5^	4.25 × 10^−2^
Q566C7	Nudt3	Diphosphoinositol polyphosphate phosphohydrolase 1	2.57 × 10^−3^	3.22 × 10^−2^

## Data Availability

The data used to support the findings of this study are included within the supplementary information file.

## Supplementary Materials

Supplementary Materials Raw data of DEPs in ISO/control group, ATH-1/ISO group, and ATH-1/control group.
